# Complex genetic architecture of the chicken *Growth1* QTL region

**DOI:** 10.1371/journal.pone.0295109

**Published:** 2024-05-13

**Authors:** Jen-Hsiang Ou, Tilman Rönneburg, Örjan Carlborg, Christa Ferst Honaker, Paul B. Siegel, Carl-Johan Rubin

**Affiliations:** 1 Department of Medical Biochemistry and Microbiology, Uppsala University, Uppsala, Sweden; 2 Virginia Polytechnic Institute and State University, School of Animal Sciences, Blacksburg, Virginia, United States of America; 3 Institute of Marine Research, Bergen, Norway; Government College University Faisalabad, PAKISTAN

## Abstract

The genetic complexity of polygenic traits represents a captivating and intricate facet of biological inheritance. Unlike Mendelian traits controlled by a single gene, polygenic traits are influenced by multiple genetic loci, each exerting a modest effect on the trait. This cumulative impact of numerous genes, interactions among them, environmental factors, and epigenetic modifications results in a multifaceted architecture of genetic contributions to complex traits. Given the well-characterized genome, diverse traits, and range of genetic resources, chicken (*Gallus gallus*) was employed as a model organism to dissect the intricate genetic makeup of a previously identified major Quantitative Trait Loci (QTL) for body weight on chromosome 1. A multigenerational advanced intercross line (AIL) of 3215 chickens whose genomes had been sequenced to an average of 0.4x was analyzed using genome-wide association study (GWAS) and variance-heterogeneity GWAS (vGWAS) to identify markers associated with 8-week body weight. Additionally, epistatic interactions were studied using the natural and orthogonal interaction (NOIA) model. Six genetic modules, two from GWAS and four from vGWAS, were strongly associated with the studied trait. We found evidence of both additive- and non-additive interactions between these modules and constructed a putative local epistasis network for the region. Our screens for functional alleles revealed a missense variant in the gene *ribonuclease H2 subunit B* (*RNASEH2B*), which has previously been associated with growth-related traits in chickens and Darwin’s finches. In addition, one of the most strongly associated SNPs identified is located in a non-coding region upstream of the long non-coding RNA, ENSGALG00000053256, previously suggested as a candidate gene for regulating chicken body weight. By studying large numbers of individuals from a family material using approaches to capture both additive and non-additive effects, this study advances our understanding of genetic complexities in a highly polygenic trait and has practical implications for poultry breeding and agriculture.

## Introduction

Phenotypic trait characteristics can be influenced by genetic and environmental factors, contributing to the unique phenotypic repertoire of each individual. Studies aimed at identifying factors influencing physical traits, including the genetic architecture of complex traits, have been carried out in the fields of medical science [1] and agriculture [2,3] for decades. These endeavors have clarified that the phenotypic segregation pattern of complex traits is different from that of quantitative traits and makes it difficult to predict with single genetic markers. Even though human Genome-Wide association studies (GWAS) are sometimes performed using hundreds of thousands of subjects, statistical power can be restrictive to identifying non-additive genetic effects on traits, such as gene-by-gene interaction (epistasis) effects. However, studies have reported significant epistasis, for example, in GWAS for human body mass index [4]. Nevertheless, we are still in the early stages of knowing how to detect epistasis with statistical approaches.

Studies have focused on critical economic traits for animals like chickens, and growth-related characteristics are one of the most crucial. These traits are known to be affected by many genes [5–8]. Despite mapping has been achieved using commercial breeds in previous studies, natural variation cannot be fully detected [6,9–11]. As a case study, we focus on an experimental chicken model system developed by divergent selection for body weight at eight weeks of age for 40 generations. Growth traits are known to be controlled by genes interacting with each other and environmental effects [12]. In our experimental system, chickens were reared in a fixed environment, limiting the extent of environmental impact on growth across generations. After 40 generations of bidirectional selection, there was a significant nine-fold difference in body weight between the high-weight (HWS) and low-weight (LWS) selected lines. Studies on the F_2_ population resulting from crosses of HWS and LWS have shown that thirteen growth-related QTLs (*Growth1*-*Growth13*) contribute to this difference. Still, they only explain a small part of the variance, indicating that many genes influence body weight [13].

From F_2_ individuals, an advanced intercross line (AIL) was established, to improve the resolution for association studies. Through multiple generations of the AIL population (F_2_-F_8_ and F_2_-F_15_), various studies have effectively accomplished the task of fine mapping and detecting selective sweeps. As generations pass and recombination accumulates, the resolution of association studies improves [14–16]. Based on parts of the now available data from the AIL population, it has been concluded that the genetic basis for 8-week body weight in Virginia lines is quite complex with presumed epistasis and higher-order interactions [17,18]. Previous studies have hypothesized that these QTLs are parts of a gene-by-gene radiation network, which may explain why they are under strong selection despite each QTL on its own having only a marginal association with weight [19]. Such a situation, where loci contributing to important epistatic interactions are not revealed in initial single marker additive screens, complicates the selection of candidate markers for epistatic scans. Meanwhile, searching for epistatic interactions genome-wide in a completely hypothesis-free manner would have quickly exhausted statistical power due to the vast number of marker-by-marker combinations needed to be tested [20].

This study investigates the genetic architecture of one specific QTL region, the *Growth1* region on chromosome 1, the most significant loci identified in previous studies performed on the Virginia lines (S1 Fig). This region was chosen as a case study for dissecting the contribution of additive- as well as non-additive genetic effects on growth. In contrast to previous work on the AIL pedigree, we now extend the scope using GWAS instead of QTL mapping. Thus, no prior assumption is used for allele frequencies in the founder lines, meaning that a larger fraction of genomic markers can be scrutinized for association. Furthermore, the current study used all individuals from the AIL pedigree generations F_2_-F_18_, for which sequencing data and phenotypes were collected. Two methods were used to identify candidate markers: genome-wide association study (GWAS) and variance-heterogeneity GWAS (vGWAS). GWAS was used to identify markers that carry mean effects in the model, which may result from additive effects. Conversely, vGWAS was used to identify markers contributing to phenotypic variance, which could be caused by epistatic interaction or haplotype effects [21]. Analysis using only GWAS could fail to expose some genetic contributions to variation in complex traits. The vGWAS methodology provides an opportunity to map incomplete linkage disequilibrium between causal polymorphisms and tested markers, multiple functional alleles, GxG interactions, and GxE interactions, which may result in heterogeneity in variance between genotypes [22–24]. Lastly, the natural and orthogonal interaction (NOIA) model was used to detect epistasis. The polygenic response to bidirectional selection combined with the multigenerational intercrossing in the AIL makes this system suitable for understanding more about the relationship between genetic and phenotypic variation resulting from haplotypes, linked loci, and epistatic interactions.

## Materials and methods

### The virginia chicken lines

#### Bidirectional selection and advanced intercross line

The Virginia Chicken Lines were established in 1957 [25–28]. The base population was produced by crossing seven mildly inbred lines of White Plymouth Rocks. From this gene pool (S_0_), they selected chickens with higher body weights at eight weeks of age (BW8) to be parents of the HWS while lighter individuals were selected to become parents of the LWS. After undergoing 40 generations of bidirectional selection, the HWS and LWS chickens exhibited a nine-fold difference in their average BW8 [13,29]. The advanced intercross line (AIL) was started by crossing HWS and LWS chickens from the 41st generation, and 17 generations of AILs were used in this study (F_2_-F_18_). Body weights of chickens at eight weeks of age were the study trait. All procedures in this study were carried out in accordance with Virginia Tech Animal Care and Use Committee protocols.

#### Sequencing and genome alignment

DNA samples from the F_0_ generation, i.e, HWS and LWS from generation 41 and their progeny F1 generation, which resulted from intercrossing HWS with LWS, were sequenced to high coverage as Illumina TrueSeq libraries on an Illumina HiSeq X instrument (SciLifeLab SNP&SEQ Technology platform, Uppsala, Sweden). We followed the Broad best practice. Reads were mapped to the reference genome galGal 6 using Burrows-Wheeler Aligner (BWA-MEM, version 0.7.17) [30]. Following alignment, duplicate reads were flagged with Picard [31]. Quality score recalibration was performed before SNP calling with HaplotypeCaller (GATK) [32–34]. Later-generation intercross samples (F_2_-F_18_) were sequenced by Illumina HiSeq 4000 with a coverage of approximately 0.4x (~0.8x for F_2_ full-sib families and ~0.4x for the remaining samples) using a segmentation and pooled library approach [35]. High to intermediate coverage depths were used for AIL F_0_ (~30x) and F_1_ (~5x). A total of 3215 chickens were used in this study. The numbers of sequenced individuals from each generation are shown in S1 Table. Custom code, software versions, and parameters can be found in our Github repository at github.com/CarlborgGenomics/AIL-scan [36] The sequencing data generated for this project can be found in the NCBI sequence read archive, BioProject PRJNA788343.

#### Imputation with pedigree information

We used pedigree-based imputation on all generations of AIL samples to obtain reliable genotypes for the low-coverage later-generation AIL individuals [37]. When pedigree information and high-coverage sequence samples are available, the inheritance of the well-characterized founder genomes to offspring can provide helpful information for imputation from ultralow-coverage sequenced data. This study includes founders sequenced at high coverage and offspring sequenced at lower coverage. We used the AlphaFamImpute software [38] which leverages pedigree information to impute and phase the genotypes of individuals. The imputation quality was tested by comparing GoldenGate data for F_15_ individuals from Sheng [16] and compared these with our imputed genotypes. The agreement for homozygous calls was 0.94, and for the heterozygous calls an agreement of 0.97 was observed (S2 Fig). A similar level of agreement was observed in a previous study of the AIL line F2 individuals [35].

### Association study

#### Genome-wide association study (GWAS)

All sequenced individuals from F_2_ to F_18_ were used in all genotype and phenotype associations performed. Using a single-marker approach, we conducted a study in R (v4.2.2) with the linear model function, “lm” function, in the “stats” package to test the association between BW8 and imputed SNP markers. The linear model we used considered sex and generation as fixed effects. In our case, we have one batch per generation every year on the same date.

$$y=1\mu +S\beta_{S}+g\beta_{g}+A_{j}a_{j}+\epsilon$$ (1)

Where *y* is the body weight vector of all chickens; *μ* is the intercept and 1 is a column vector of ones; *S* is a design matrix of sex effect with effect size *β*_*S*_; *g* is the design matrix of generation effect with the effect size vector *β*_*g*_; *a*_*j*_ is the effect size of marker *j*; and the genotype vector *A*_*j*_ is coded as the number of alternative alleles of marker *j* for each sample.

#### Variance-heterogeneity GWAS (vGWAS)

By using vGWAS analysis, we could efficiently map quantitative trait loci to variance heterogeneity (vQTL), which can be influenced by epistasis. This helps us identify additional associations between markers and traits. The Brown-Forsythe test is a statistical method used to compare the variance between groups. It involves analyzing the variance of a transformed response variable using ANOVA. For sample *i* with genotype *j*, the absolute deviation from the median body weight of each genotype group is shown in the following equation.

$$y_{ij}^{*}=\left|y_{ij}-\varphi_{j}\right|$$ (2)

Where *φ*_*j*_ is the median body weight of sample with genotype *j*. The Brown-Forsythe test statistic is the F value of the ANOVA on the absolute deviation $y_{ij}^{*}$. Similar to the GWAS model, it is important to account for sex and generation effects, so we normalized phenotype values within each sex-generation group before the analysis.

#### Haplotype-based association study

Haplotype-based association analysis should be more robust than single-marker analysis because the former utilizes information about linkage disequilibrium (LD) from multiple markers [39,40]. The model, Eq (3), used in our study was similar to the one used for GWAS. The haplotype effect replaced the marker effect while considering sex and generation as covariates.


$$y=1\mu +S\beta_{S}+g\beta_{g}+H_{j}h_{j}+\epsilon$$ (3)

Plink was used to determine haplotype blocks using the default setting across genotypes in all AIL samples [41–43].

#### Ancestry haplotype painting and association study

The main ancestry contributors of current White Plymouth Rock chickens were Dominique, Black Java, and Cochin, while Brahmam, Langshan, and Black Minorca have lesser contributions [44]. We used the ChromoPainter software [45] to identify haplotypes most likely inherited from specific ancestor breeds. We then performed the haplotype-based association study with Eq (3) using haplotypes classed by ancestor breed of origin.

### Detection of epistasis

Epistasis is the situation in which the effect of an allele in a locus is modified by the presence or absence of an allele in another locus. Therefore, this effect can be identified by stratifying the available data set by genotype at one candidate epistasis locus and then comparing the effect of another locus (or several loci). The natural and orthogonal interaction (NOIA) model was first applied to identify statistical evidence of epistasis. After that, for each target marker selected from GWAS and vGWAS, we stratified samples into three subsets by the genotype of the target marker.

#### The NOIA model

The NOIA model was developed to estimate the main and interaction effects among loci while adjusting for unbalanced allele frequencies [46]. Statistically, gene effects remain orthogonal and provide consistency in reducing models. For a two-loci model (loci A and B) having a genetic effect vector *E*_*AB*_ with a design matrix *S*_*AB*_, the genotype value *G*_*AB*_ can be expressed by the following equation.


$$G_{AB}=S_{AB}\cdot E_{AB}=\left(S_{B}⊗S_{A}\right)\cdot E_{AB}$$ (4)

Therefore, the genetic effect can be estimated by Eq (5)

$$E_{AB}=\left(S_{B}^{-1}⊗S_{A}^{-1}\right)\cdot G_{AB}$$ (5)

However, this model is insufficient for describing the genetic effects from any given reference point. To extend this, derivation accounts for genotyping frequencies (*p*_11_, *p*_12_, and *p*_22_). Considering a one-locus mode, the design matrix *S*_*F*_ can be expressed as Eq (6) with the reference point *R* = *p*_11_ *G*_11_ + *p*_12_ *G*_12_ + *p*_22_ *G*_22_.


$$S_{F}=\left(1\;-p_{12}-2p_{22}\;-p_{12}\;1\;1-p_{12}-2p_{22}\;1-p_{12}\;1\;2-p_{12}-2p_{22}\;-p_{12}\right)$$ (6)

Therefore, the two-loci model can be easily reached by replacing *S*_*A*_ and *S*_*B*_ in the Eq (4) with two design matrices in Eq (6). This analysis was performed by the NOIA package (version 0.97.3) in R.

### Screening for functional variants

Candidate genes were searched within chromosome 1 150-180Mb area by including association study results, sequence ontology, and evolutionary constraint information. Sequence ontology terms (GRCg6a.105) were annotated to the VCF file by snpEff [47,48]. Conservation scoring by phylogenetic *P*-values (PhyloP score) from the PHAST package for multiple alignments of 76 genomes to the chicken genome were downloaded from the UCSC database (galGal6/phyloP77way). Candidates are those markers that have low *P*-values and high conservation scores. Minor allele frequencies are considered to avoid increasing false discovery rates in association studies.

## Results

### Genome-wide association study (GWAS)

**Fig 1:**
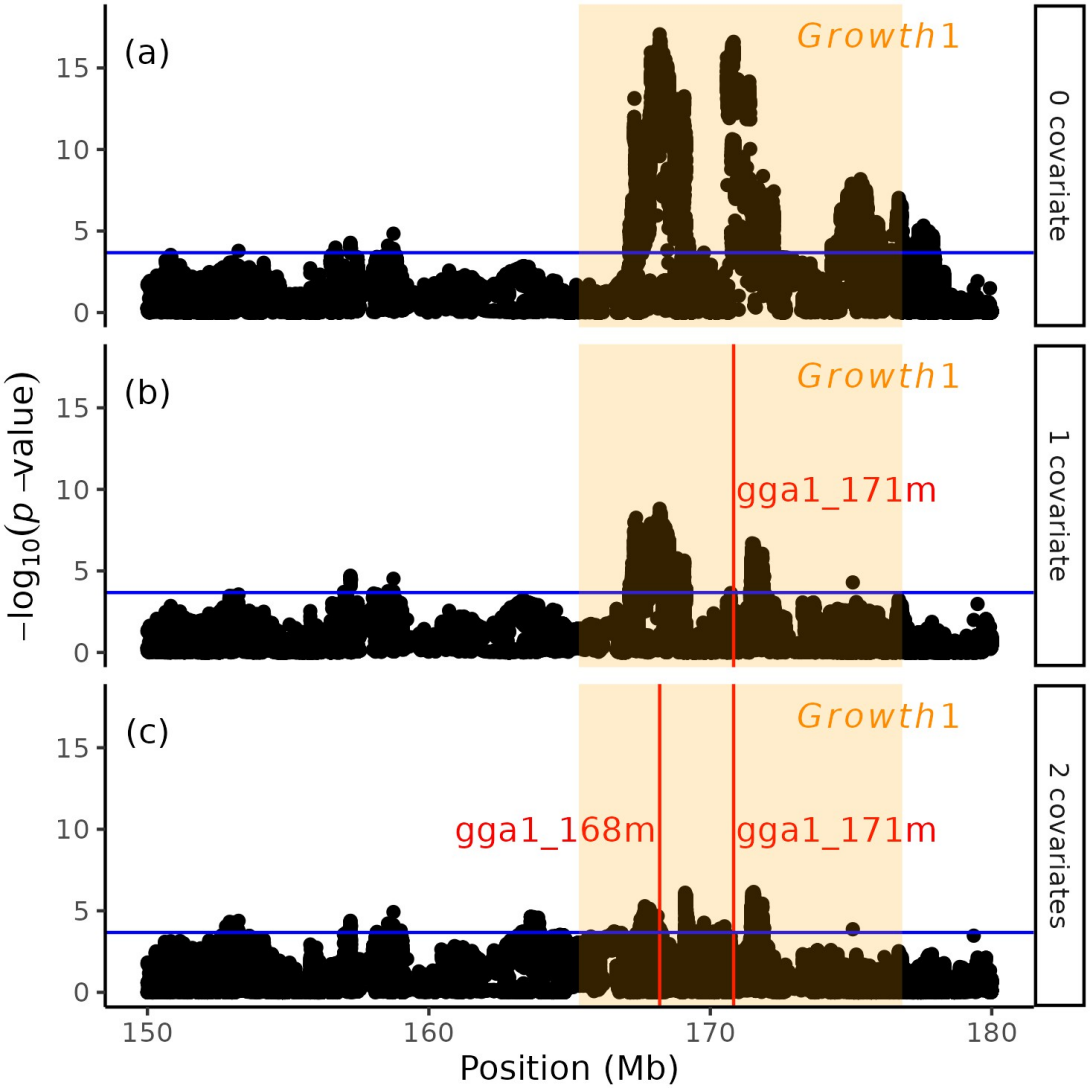
GWAS results on chromosome 1 150-180Mb region. (a) Result of standard GWAS. Figures (b) and (c) show the result of adding the top SNP markers as covariates. The result shows that after adding the right peak as a covariate, the left peak signal remains moderate. This implies that both peaks could carry different effects. The QTL Growth1 region (chr1:165330388–176818938) is annotated with an orange translucent mask.

**Fig 2:**
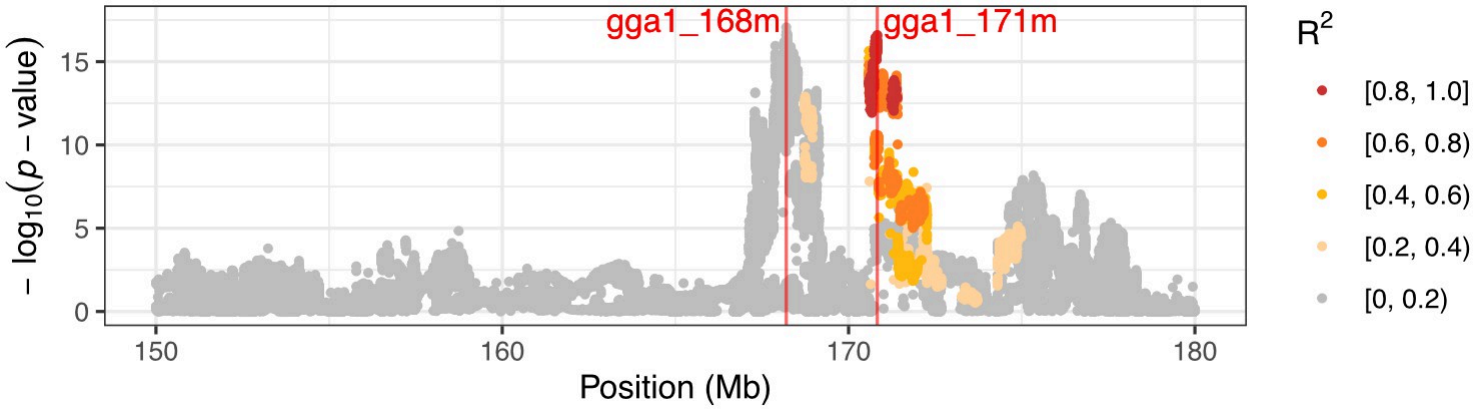
GWAS results and linkage disequilibrium (LD) on chromosome 1 150-180Mb region. LD was painted related to the marker gga1_171m. Variants in the gga1_168m peak do not show high LD to marker in the gga1_171m.

A genome-wide association study (GWAS) was first performed to determine the top SNP markers in the previously known QTL *Growth1*. As shown in Fig 1a, two significant peaks, gga1_168m (chr1:168200669) and gga1_171m (chr1:170731384), were detected, which was also the case in a previous study [37]. To study if these two signals were statistically independent, the right peak top SNP, gga1_171m, was considered a covariate to the GWAS model (Fig 1b). Adding the gga1_ 171m SNP marker as a covariate, the significance of the left-hand peak declined while not vanishing. Furthermore, no peaks were detected in the region when we used both significant peaks as covariates in the model (Fig 1c). To explain the complex genetic architecture of this region, we provide two possible explanations. First, there could be distinct haplotype effects due to LD between the functional alleles that are not captured by individual SNP markers. The LD in the region shows that gga1_171m and gga1_168m markers are not strongly linked (Fig 2). Second, interactions between the loci result in nonadditive genetic variance not explained in the additive model but which, instead, we hypothesize could be captured as genetic variance heterogeneity.

### Variance-heterogeneity genome-wide association study (vGWAS)

**Fig 3:**
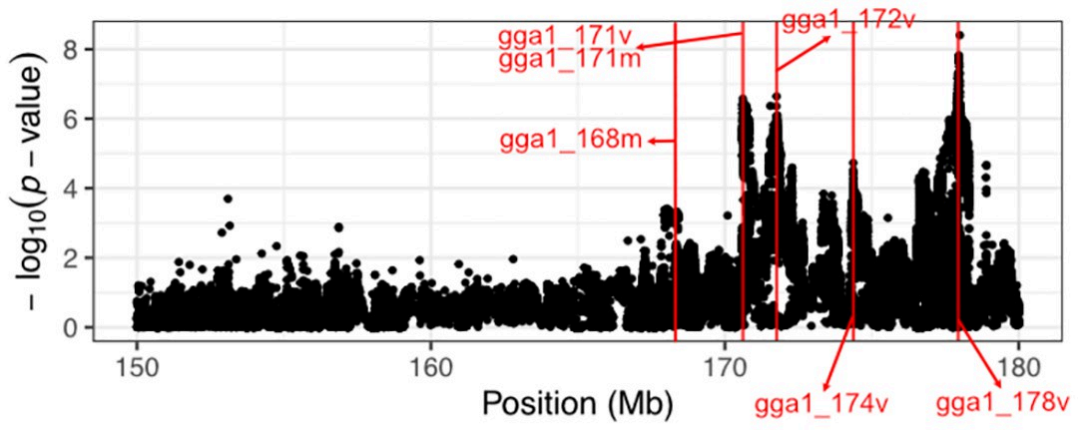
Variance-heterogeneity GWAS result. Red lines annotate the position of selected SNPs showing variance effects.

**Fig 4:**
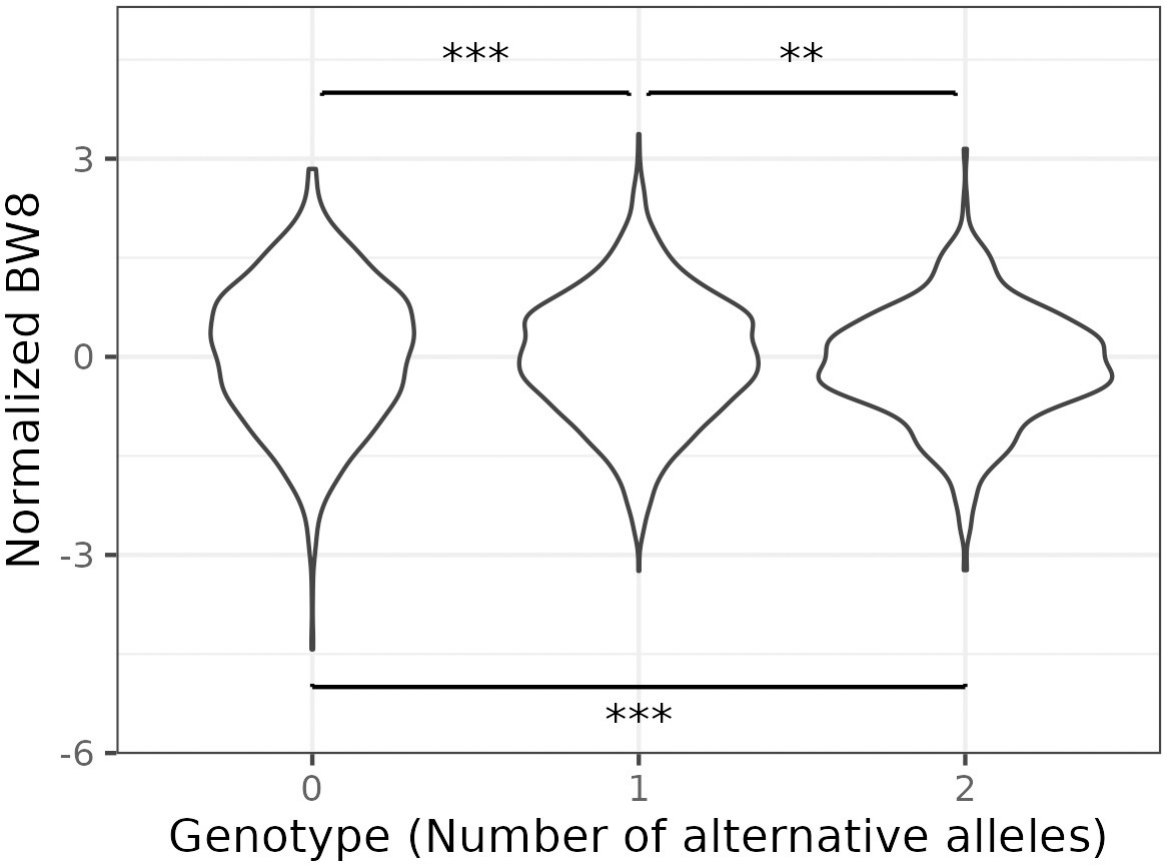
Distributions of normalized body weight stratified by genotype at marker gga1_178v. Violin plots showing variance differences between genotype groups. The star signs show the pairwise significance of the variance effect.

To investigate the complex genetic architecture of Growth1 QTL, we performed a vGWAS to screen for SNP markers carrying variance effects. Such markers could represent loci contributing to genetic interactions [21]. This analysis identified four additional signals downstream of standard GWAS peaks (Fig 3). Selected SNP markers are gga1_171v (chr1:170613341), gga1_172v (chr1:171761454), gga1_174v (chr1:174370270), and gga1_178v (chr1:177940599). Individual phenotypic means by genotype groups at gga1_178v are shown in Fig 4.

### Haplotype effect

**Fig 5:**
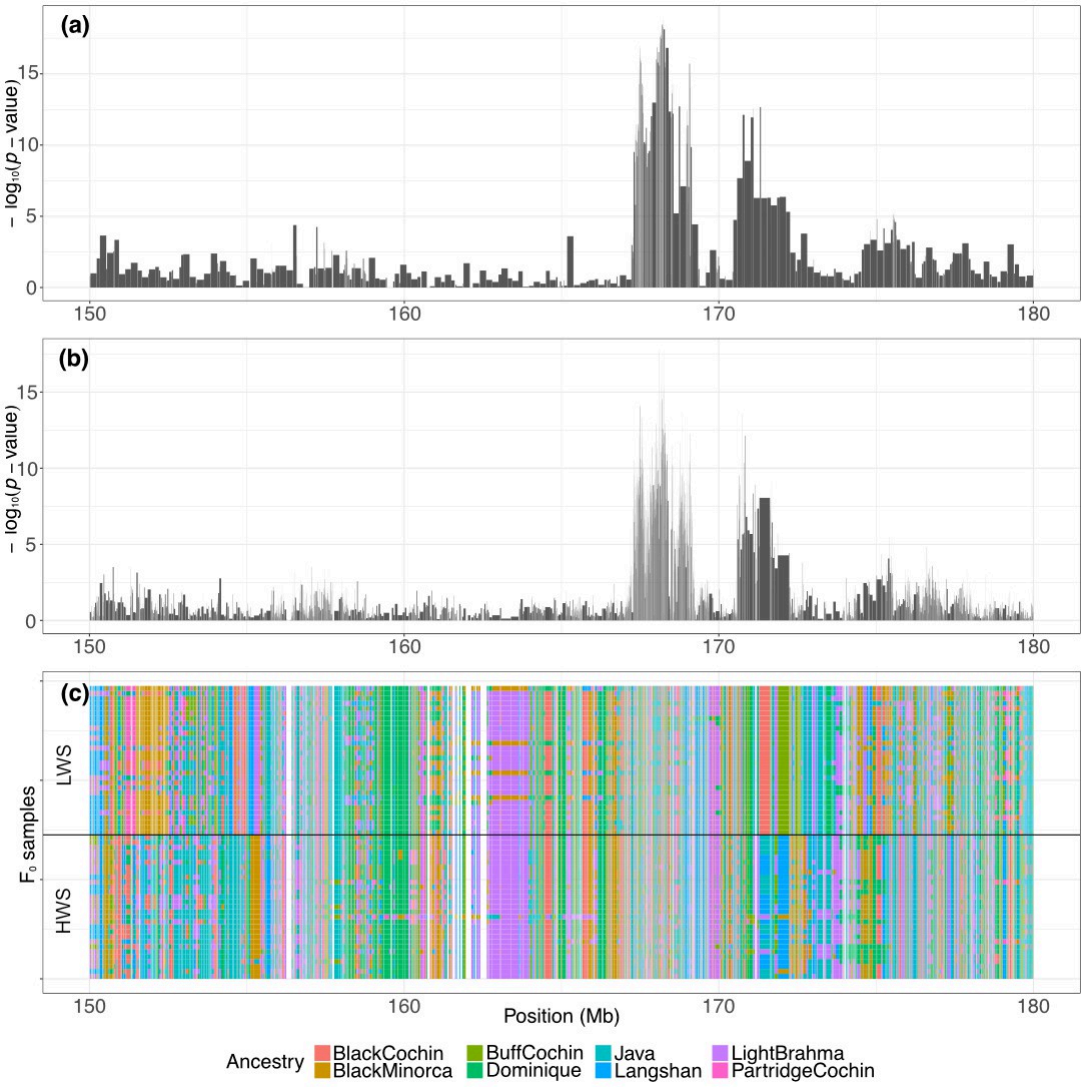
Haplotype-based association study. Haplotype-based association study results on chromosome 1 150-180Mb region. (a) General haplotype-based association study. A negative log of the *P*-value on the y-axis shows significant results. (b) Ancestry haplotype association study result. (c) Haplotype mosaic plot for F_0_ generation of the population. Each row represents a sample. The color stands for different ancestry donors.

As discussed in the previous section, the haplotype effect is the first possible explanation for causing the complex genetic architecture of *Growth1*. In Fig 2, it can be seen that the LD between the functional alleles is not captured by individual SNP markers. This indicates that multiple loci contribute to the body weight trait. We conducted a haplotype-based GWAS analysis to validate the hypothesis that two peaks have independent effects, revealing that the two previously detected GWAS peaks coincided with those obtained in a haplotype-based association study (Fig 5a). Additionally, there still exists a region of non-association between the two peaks. This suggests that two peaks are less likely to exist on the same haplotype.

To further understand the origin of that haplotype, both HWS and LWS samples were painted by ancestry information [44]. The ancestral haplotype association study yielded similar results to the haplotype-based association study, with the two main GWAS peaks showing strong significance, as seen in Fig 5b and 5c, where the latter shows ancestral haplotype blocks. It was discovered that most of the LWS samples were fixed for one haplotype in the right peak, corresponding with earlier studies on the haplotype complexity in the *Growth1* region with the bi-directional selected lines [17]. That study revealed that the 55th generation of LWS samples had only one LWS haplotype in this region. Conversely, HWS samples contained various haplotypes [17]. For the left peak (gga1_168m), three major haplotypes were identified. The highest frequency haplotype was present in both HWS and LWS samples but had a higher frequency in the LWS, while the other two haplotypes existed only in either HWS or LWS samples, with the statistical model estimating that they have significantly different effects of 26 and -3 grams, respectively.

### Independent marker effect

The independent marker effects were tested using a stepwise selection across determined SNP markers. Sex and generation were considered fixed effects in the model. Eventually, gga1_168m, gga1_171v, and gga1_178v remain in the final model (Table 1).

**Table 1:** Testing results of the linear model fitted with markers selected by stepwise regression.

Effects	Mean Square	F value	*P*-value
Sex	1.47 × 10^7^	689.47	< 0.001***
Generation	1.32 × 10^6^	61.72	< 0.001***
gga1_168m	1.51 × 10^6^	70.80	< 0.001***
gga1_171v	6.34 × 10^5^	29.65	< 0.001***
gga1_178v*	5.68 × 10^4^	2.66	0.1032

Significant code: 0 ‘***’ 0.001 ‘**’ 0.01 ‘*’ 0.05 ‘.’ 0.1 ‘ ‘ 1.

* gga1_178v was included in stepwise regression and was strongly significant in the vGWAS while not significant after adding covariates in the model.

We calculated the average body weight and standard deviation to evaluate how the mean effect changed with the number of reference or alternative alleles (Table 2). The least significant difference (LSD) analysis was used to test if there was a significant difference in body weight among genotype groups. For each of the loci gga1_168m, gga1_171m, gga1_174v, and gga1_178v, body weight decreased with increasing numbers of alternative alleles. For gga1_168m, the effect size was relatively large, while the numbers of individuals in each group were not balanced (1691 in the RR group vs. 164 in the AA group). For gga1_171m and gga1_174v numbers of individuals in opposite homozygote groups were more balanced. For gga1_171v and gga1_172v, average body weight increased with the number of alternative alleles.

**Table 2:** Average body weight grouped by the genotype of the target marker. Genotype coding is given by reference (R) and alternative (A) alleles. Body weight was normalized within the generation-sex groups to remove effects that we were not interested in. The last column presents the LSD result by grouping notations a to c (significance level *α* = 0.05).

Target	Genotype^1^	Average BW^2^	STD^3^	Count^4^	LSD^5^
gga1_168m	RR	0.1194	0.9893	1691	a
	RA	-0.1183	0.9861	1075	b
	AA	-0.4452	0.8843	164	c
gga1_171m	RR	0.1979	1.0384	692	a
	RA	0.0010	1.0069	1552	b
	AA	-0.2019	0.8765	686	c
gga1_171v	RR	-0.1751	0.9158	989	a
	RA	0.0375	1.0125	1538	b
	AA	0.2865	1.0331	403	c
gga1_172v	RR	-0.1306	0.8909	705	a
	RA	0.0004	1.0117	1574	b
	AA	0.1405	1.0418	651	c
gga1_174v	RR	0.0770	1.0208	939	a
	RA	-0.0050	1.0139	1509	b
	AA	-0.1343	0.8617	482	c
gga1_178v	RR	0.0638	1.0719	1071	a
	RA	-0.0053	0.9631	1426	a
	AA	-0.1404	0.8803	433	b

^1^ The frequencies of reference and alternative alleles in the HWS and LWS population is shown in S2 Table.

^2^ Average normalized body weight.

^2^ Standard Deviation of average normalized body weight.

^3^ Number of chickens in each group.

^4^ Least significant difference. Genotype groups having a different letter have a statistically significant difference in body weight.

### The NOIA model

**Fig 6:**
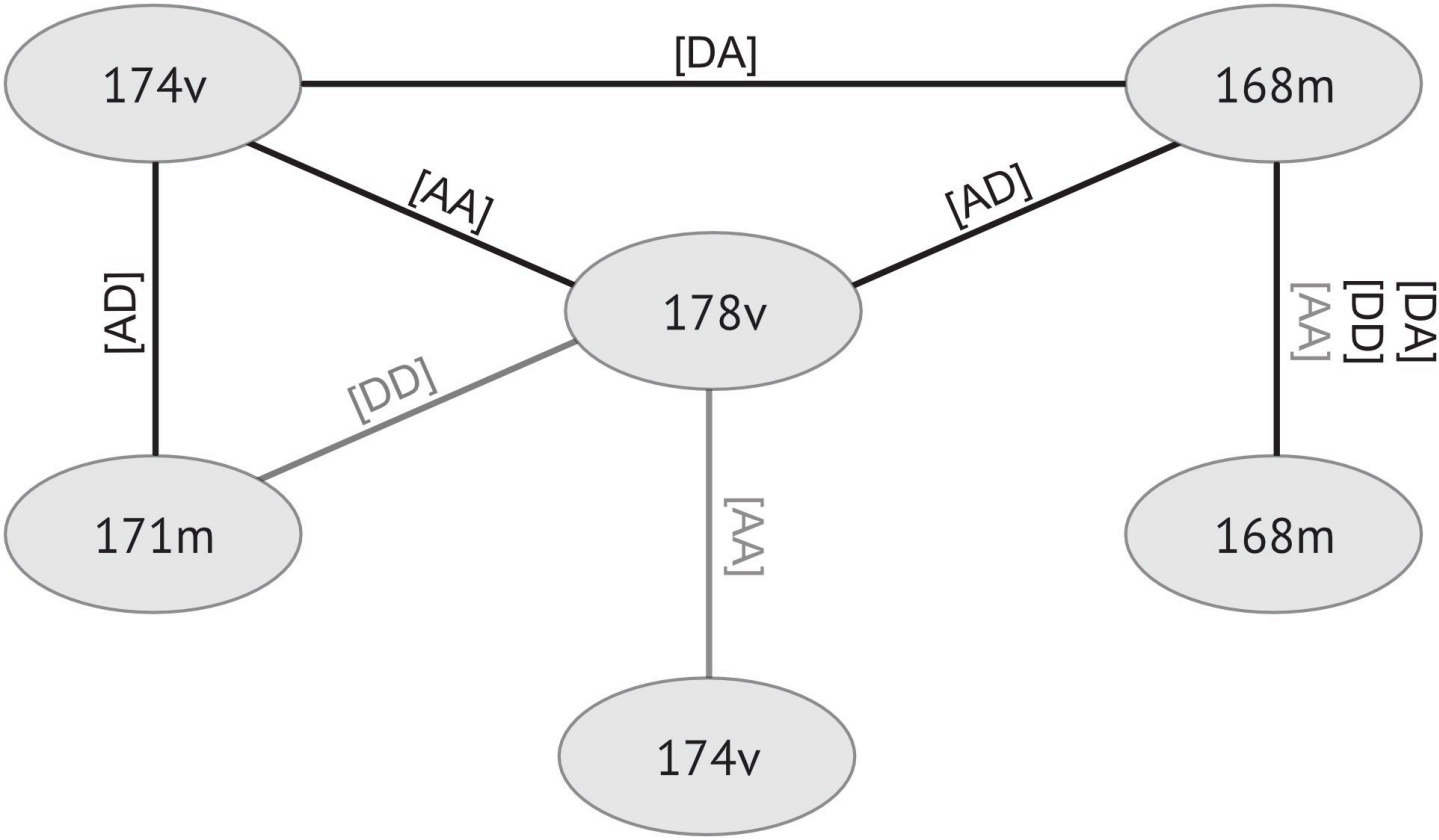
Significant interaction effect among selected markers. A and D stand for additive effects and dominance effects. Line colors indicate the degree of statistical significance, with darker colors indicating lower *P*-values.

The NOIA model fits six markers selected from both GWAS and vGWAS results (S3 Table). All additive, dominance, and second-order interaction effects are included in the model. Sex and generation effects are removed by normalizing the phenotype within each sex-generation group. Fig 6 shows all significant second-order interactions among selected markers. Three markers obtained highly significant interaction effects by NOIA, and these are positioned in the upper triangle of the network, which includes gga1_168m, gga1_174v, and gga1_178v. Markers gga1_171m and gga1_172v showed significant interaction with gga1_174v and gga1_168m, respectively. The last marker, gga1_171v, had a mild additive interaction effect with gga1_178v. The significant interactions shown in Fig 6 support that regulation of body weight by *Growth1* QTL cannot be described simply by independent effects of the loci.

### Epistasis effects

The NOIA model result shows that several interactions among selected SNP markers are highly associated with body weight. The average and standard deviation of body weight grouped by genotypes of two selected markers shows how mean and variance change with the interaction between two markers. Association analysis was performed with samples grouped by genotypes of the target markers to observe significant changes under the condition of different genotypes (S3–S7 Figs).

**Fig 7:**
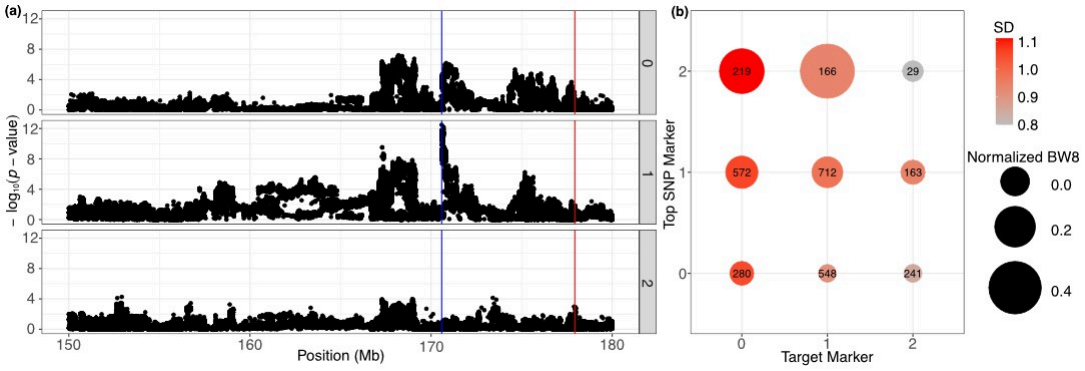
Epistasis effects conditioned on gga1_178v. (a), samples were grouped by genotype of gga1_178v (red vertical line), in which 0, 1, and 2 represent the number of alternative alleles. The top SNP marker is annotated by the blue vertical line. (b) shows the normalized average and standard deviation body weight in different conditions.

The two GWAS peaks, gga1_168m and gga1_170m, remained strong in individuals carrying at least one gga1_178v reference allele, which has a higher frequency in the HWS samples (two upper panels of Fig 7a). However, both gga1_168m and gga1_170m signals were absent in alternative allele homozygotes at gga1_178v. Fig 7b gave the same suggestion, while we group samples by the genotype of the top SNP marker (blue vertical line) and gga1_178v, body weight has a minor difference between alternative allele homozygotes at gga1_178v. In contrast, samples carrying at least one reference allele show a significant difference between groups. Markers gga1_172v (S6 Fig) and gga1_174v (S7 Fig), on the other hand, show a different pattern. Two GWAS peaks were eliminated in both homozygous groups.

### Candidate causal genes

To screen associated loci for protein coding and UTR effects, we annotated variants by snpEff (GRCg6a.105). In addition, we intersected variants with vertebrate PhyloP scores to be able to prioritize associated variants in non-coding regions based on evolutionary constraints. In S8 Fig, SNP markers are colored by their snpEff classification categories and plotted by increasing GWAS *P*-values and PhyloP scores. Minor allele frequency information was added to ensure the statistical significance is not due to the unbalanced data structure. Markers with minor allele frequencies greater than 0.1 and PhyloP scores ranking in the top 5% were sorted by the *P*-value and are presented in S4 Table. None of the SNP markers showing most strongly associated with body weight were predicted to change coding parts of genes (Tables 3 and S4), and were clustered in introns of or intergenic to the genes *Ecto-NADPH Oxidase Disulfide-Thiol Exchanger 1* (*ENOX1*), ENSGALG00000050514, ENSGALG00000052226 ENSGALG00000053256. Interestingly, ENSGALG00000053256, a novel long non-coding RNA has previously been implicated as a candidate gene for regulating chicken body weight [49]. Several strongly associated SNP variants were predicted to cause amino acid substitutions in genes (Tables 3 and S5). Of these the protein encoded by *TNF superfamily member 11* (*TNFSF11*) has known effects on bone growth and *ribonuclease H2 subunit B* (*RNASEH2B*) has previously been implicated as a candidate gene for body weight in chicken [52].

**Table 3:** The top associated overall- and missense-variants. Refer to the supplementary files, S4 and S5 Tables, for accessing a more complete table.

POS	REF	ALT	GWAS^1^	PhyloP	AAF^2^	Effect	GeneName	Transcript change	Protein change
**Overall variants**									
168203470	A	T	16.61	2.84	0.23	intron_variant	*ENOX1*	-	-
168203263	A	C	16.61	1.72	0.23	intron_variant	*ENOX1*	-	-
170835310	A	G	16.22	1.32	0.48	upstream_gene_variant	*ENSGALG00000050514*	-	-
170835310	A	G	16.22	1.32	0.48	intergenic_region	*ENSGALG00000050514- ENSGALG00000053256*	-	-
168216902	G	T	16.21	1.22	0.22	intron_variant	*ENOX1*	-	-
170812758	T	C	16.17	1.24	0.5	intron_variant	*ENSGALG00000050514*	-	-
170808018	T	C	16.06	2.58	0.49	intron_variant	*ENSGALG00000050514*	-	-
168211327	A	G	16.01	1.37	0.22	intron_variant	*ENOX1*	-	-
168228062	A	G	15.98	1.41	0.23	intron_variant	*ENOX1*	-	-
168223991	T	C	15.89	2.12	0.23	intron_variant	*ENOX1*	-	-
***Missense variants***									
170685654	G	T	14.93	1.3	0.38	missense_variant	*SPRYD7*	c.47C>A	p.Ala16Asp
169127401	C	G	10.32	3.74	0.21	missense_variant	*COG3*	c.1489C>G	p.Gln497Glu
167844491	T	C	9.44	2.14	0.19	missense_variant	*TNFSF11*	c.281T>C	p.Ile94Thr
168961702	C	T	8.3	1.99	0.28	missense_variant	*GPALPP1*	c.440C>T	p.Thr147Ile
168962167	A	G	8.15	3.26	0.28	missense_variant	*GPALPP1*	c.677A>G	p.Lys226Arg
171246434	A	G	7.57	4.43	0.43	missense_variant	*RNASEH2B*	c.470A>G c.467A>G	p.Tyr157Cys p.Tyr156Cys
171405714	G	A	7.29	3.25	0.43	missense_variant	*SERPINE3*	c.530G>A	p.Arg177Gln
171411240	C	T	7.22	2.6	0.43	missense_variant	*SERPINE3*	c.920C>T	p.Ala307Val
167751114	G	A	6.23	3.84	0.19	missense_variant	*AKAP11*	c.5336G>A	p.Ser1779Asn
175921491	A	G	5.06	1.3	0.13	missense_variant	*BRCA2*	c.4879T>C	p.Tyr1627His

^1^ -log(10) GWAS *P*-value.

^2^ Frequency of the alternative allele.

## Discussion

Gallus gallus autosomal chromosome 1 (GGA1) contains one known QTL, *Growth1*, which includes two strongly associated peaks confirmed by studies using early generations of the AIL population [14,37]. With more generations included, recombination accumulation will increase the resolution of association studies. Our study focused on an extended region on chromosome 1 (chr1:150–180 Mb), including the *Growth1* peak (chr1:165.3–176.8 Mb).

This study advanced the fine mapping of body weight-related variants in chickens. In previous research, suggestive QTL regions [13,14,50–52] and epistasis between QTLs [15,19,53] were consistent with the thesis that in chickens, body weight is highly polygenic, moderate to highly heritable, and influenced by non-additive effects. However, previous studies did not fully consider local epistasis. Because recombination accumulates with each additional generation of the AIL population, we have a higher resolution than our previous association studies, which used only parts of the genetic data that are now available for the AIL. Here, we focus on one of the suggestive QTLs, the *Growth1* region, and describe a complex genetic architecture within the region.

Haplotype effects may be considered major effectors of the complex genetic architecture observed in the *Growth1* region. When we added two top SNP markers selected by GWAS as covariates, they explained most variants, and the remaining significance signal was weak. Forward selection and backward elimination also supported that the three selected markers were a better combination for explaining observed phenotype differences. As shown by Guo et al. [44], the admixture process involved in developing the White Plymouth Rock breed can, 150 years later, be used to trace haplotype breeds of origin in HWS and LWS lineages. To evaluate the potential of using the ancestral information, and hence the historical recombination that has occurred, in the fine mapping of the QTL in the Virginia AIL population, we explored the mosaic of the lines using this breed formation event as the reference of analysis. The ancestral haplotype analysis provided support to the haplotype analysis. Both analyses resulted in consistent association results where haplotypes close to two selected GWAS peaks were significantly associated with body weight. As mentioned in Guo’s study [44], 89% of the autosomal genome was from 4 major donors (Dominique, Buff Cochins, Partridge Cochins, and Black Java); the rest of them were donated by Light Brahma (4% in HWS and 7% in LWS) and Langshan (7% in HWS and 4% in LWS). In the left peak (gga1_168m) of the *Growth1* region, most samples carried the most common ancestral haplotype, and we found a significant difference in body weight between samples that carried either the second (26 g) or third (-3 g) most common ancestral haplotypes that could only be found in HWS and LWS, respectively.

To evaluate putative intra-chromosomal interactions between loci and haplotypes significantly associated with body weight and its variance, we screened for epistasis. NOIA is a model with the benefit of orthogonality, and variant effects can be easily estimated by a given reference point. All additive, dominance, and interaction effects lower than second order were added to the model. If performed genome-wide, because of multiple testing, adding many coefficients could drastically lower the significance of NOIA results. Thus, we chose a small number of significant loci for inclusion in the model. The NOIA model provides another way of explaining the architecture in this region, which was found to be a radiation network with the gga1_178v marker sitting in the center (Fig 6). Epistasis was further explored by pairwise grouping of individuals by their genotypes at each conditioned marker. Markers detected in vGWAS analysis appear to modulate the gene-by-gene effect of gga1_168m and gga1_171m on body weight. GWAS signals were eliminated, while gga1_178v is homozygous in the reference genome, and the reference genome has a higher frequency in the LWS samples. In contrast, gga1_172v and 174v eliminate GWAS signals while having a homozygous genotype in either reference or alternative alleles.

Using association *P*-values, gene annotation by the software snpEff and evolutionary constraint information, we screened associated variants for those most likely to be causal of the observed GWAS associations. As seen in S8 Fig, the lowest *P*-value SNPs occur in the non-coding category, where several of the markers with the lowest *P*-values also show strong evolutionary constraints. It is likely that this set harbors one or several causal alleles underlying the effects of the *Growth1* QTL.

The gene *ribonuclease H2 subunit B* (*RNASEH2B*) (chr1:171220990–171264767) within *Growth1* has been implicated as a candidate gene in a GWAS for growth performed in an F_2_ pedigree from an intercross between fast-growing broiler and slow-growing Chinese indigenous breeds [52]. In that study, *RNASEH2B* was the second closest gene to the top marker. Furthermore, *RNASEH2B* recently emerged as the main candidate gene for a GWAS peak regulating beak size and shapes in Darwin’s finches [54]. *RNASEH2B* encodes one of two non-catalytic subunits of RNAse H2, an RNAse thought to play a role in DNA replication, which removes ribonucleotides from DNA to maintain genomic integrity and is mutated in the human neuroinflammatory syndrome Aicardi-Goutieres syndrome type 2 [55,56]. We found one highly associated, strongly conserved missense variant (rs737861556) in *RNASEH2B* (S9 Fig, Table 3) which is predicted to change a strongly evolutionary conserved Tyrosine to a Cysteine at amino acid position 156 whereas Tyr is almost exclusively observed across birds, mammals, reptiles and amphibians. The repeated implications of *RNASEH2B* with growth-related phenotypes in birds make it a promising candidate gene, but the exact mechanisms by which the identified amino acid change in RNAse H2 may control growth are obscure based on the known functions of this gene.

One of the most strongly associated variants we observed (chr1:170835310) (Table 3) was located 62 kb upstream of the long non-coding RNA ENSGALG00000053256, which was recently identified as one of the top candidate genes for controlling growth traits in chicken by intersections of ATAC-sequencing peaks with growth GWAS data [49]. Notably, several of the most strongly significant variants observed in our study clustered to introns of *ENOX1*, whose gene product is involved in plasma membrane transport pathways, but to our knowledge, this gene has not previously been linked directly with growth traits. In order to go beyond the results provided here, which are solely based on genetics, and to ultimately pinpoint causal variants in the *Growth1* region, we suggest that functional genomics assays should be employed. Such assays could include thorough RNA sequencing, single-cell RNA sequencing, in vitro reporter assays, ATAC-sequencing and Hi-C or Capture-C to investigate gene expression, regulatory potential of variants and chromatin interactions within the region for different genotype groups.

## Supporting information

S1 Table Number of sequenced samples in each generation. Body weight information for the F1 population was not measured. From F2 to F18, individuals with bodyweight measurements were counted. Average body weight and its standard deviation are presented in grams.
(PDF)

S2 Table Allele frequencies in the HWS and LWS samples. This table shows reference (RAF) and alternative (AAF) allele frequencies calculated in the HWS and LWS populations.
(PDF)

S3 Table Significant effects of the NOIA model. A and D represent additive and dominance effects.
(PDF)

S4 Table Candidate markers. Top 30 markers out of 1029 that passed the threshold. These markers were sorted based on their GWAS *P*-value and had a minimum MAF of 0.1 and a PhyloP score in the top 5% across the genome.
(PDF)

S5 Table Missense variants. Missense variants with the top 5% PhyloP score were sorted by GWAS significance
(PDF)

S1 Fig Genome-wide association study. The y-axis shows the significance of the association study by negative log *P*-value. The strongest significance signals lay in the *Growth1* region.
(PDF)

S2 Fig Agreement between GoldenGate assay and imputed genotype. The result shows a 97% average agreement of heterozygous and 94% agreement of homozygous between GoldenGate assay and imputed genotype of F15 samples.
(PDF)

S3 Fig Epistasis effects conditioned on gga1_168m. Figure (a), samples were grouped by the genotype of gga1_168m (red vertical line), in which 0, 1, and 2 represent the number of alternative alleles. Figure (b) shows the normalized average and standard deviation body weight in different conditions. The top SNP marker is annotated by the blue vertical line in Figure (a).
(PDF)

S4 Fig Epistasis effects conditioned on gga1_171m. Figure (a), samples were grouped by the genotype of gga1_171m (red vertical line), in which 0, 1, and 2 represent the number of alternative alleles. Figure (b) shows the normalized average and standard deviation body weight in different conditions. The top SNP marker is annotated by the blue vertical line in Figure (a).
(PDF)

S5 Fig Epistasis effects conditioned on gga1_171v. Figure (a), samples were grouped by the genotype of gga1_171v (red vertical line), in which 0, 1, and 2 represent the number of alternative alleles. Figure (b) shows the normalized average and standard deviation body weight in different conditions. The top SNP marker is annotated by the blue vertical line in Figure (a).
(PDF)

S6 Fig Epistasis effects conditioned on gga1_172v. Figure (a), samples were grouped by the genotype of gga1_172v (red vertical line), in which 0, 1, and 2 represent the number of alternative alleles. Figure (b) shows the normalized average and standard deviation body weight in different conditions. The top SNP marker is annotated by the blue vertical line in Figure (a).
(PDF)

S7 Fig Epistasis effects conditioned on gga1_174v. Figure (a), samples were grouped by the genotype of gga1_174v (red vertical line), in which 0, 1, and 2 represent the number of alternative alleles. Figure (b) shows the normalized average and standard deviation body weight in different conditions. The top SNP marker is annotated by the blue vertical line in Figure (a).
(PDF)

S8 Fig SNPs on chromosome 1 150-180Mb region colored by sequence ontology terms. The y-axis shows the PhyloP score, and the x-axis shows the significance of the GWAS result. The dot size indicates the minor allele frequency.
(PDF)

S9 Fig Markers nearby *RNASEH2B* gene. Markers simultaneously satisfy the top 5% GWAS and PhyloP score threshold near the *ribonuclease H2 subunit B* (*RNASEH2B*) gene (annotated by orange background).
(PDF)
