# Supplementary material for: Complex genetic architecture of the chicken Growth1 QTL region
Source: PLoS One. 2024 May 13;19(5):e0295109. doi: 10.1371/journal.pone.0295109 (PMC11090294; doi:10.1371/journal.pone.0295109)
Supplement: S1 Table — Body weight information for the F1 population was not measured. From F2 to F18, individuals with bodyweight measurements were counted. Average body weight and its standard deviation are presented in grams. (PDF) [file pone.0295109.s001.pdf]

***S1 Table.*** *Number of sequenced samples in each generation. Body weight information for the F1 population was not measured. From F2 to F18, individuals with bodyweight measurements were counted. Average body weight and its standard deviation are presented in grams.*

| Generation | Count | Average (SD) g |
|---|---|---|
| F0 (HWS) | 29 | 2533 (36) |
| F0 (LWS) | 30 | 181 (5) |
| F1 | 82 | N/A |
| F2 | 766 (734+32)* | 630 (187) |
| F3 | 368 | 695 (167) |
| F4 | 110 | 595 (131) |
| F5 | 117 | 656 (159) |
| F6 | 88 | 769 (174) |
| F7 | 48 | 728 (181) |
| F8 | 204 | 391 (127) |
| F9 | 79 | 728 (173) |
| F10 | 83 | 649 (172) |
| F11 | 80 | 582 (166) |
| F12 | 76 | 603 (180) |
| F13 | 88 | 628 (182) |
| F14 | 84 | 750 (190) |
| F15 | 653 | 592 (146) |
| F16 | 89 | 670 (171) |
| F17 | 41 | 502 (125) |
| F18 | 85 | 539 (152) |

* Two separate batches of F2 individuals were bred, one larger for the initial QTL-mapping and a smaller one as founders for the later generations
