# Supplementary material for: Complex genetic architecture of the chicken Growth1 QTL region
Source: PLoS One. 2024 May 13;19(5):e0295109. doi: 10.1371/journal.pone.0295109 (PMC11090294; doi:10.1371/journal.pone.0295109)
Supplement: S2 Table — This table shows reference (RAF) and alternative (AAF) allele frequencies calculated in the HWS and LWS populations. (PDF) [file pone.0295109.s002.pdf]

S2 Table. *Allele frequencies in the HWS and LWS samples. This table shows reference (RAF) and alternative (AAF) allele frequencies calculated in the HWS and LWS populations.*

	RAF in HWS	AAF in HWS	RAF in LWS	AAF in LWS
gga1_168m	1	0	0.83	0.17
gga1_171m	0.29	0.71	1	0
gga1_171v	1	0	0	1
gga1_172v	0.19	0.81	1	0
gga1_174v	1	0	0.08	0.92
gga1_178v	1	0	0.3	0.7
