# Supplementary material for: Complex genetic architecture of the chicken Growth1 QTL region
Source: PLoS One. 2024 May 13;19(5):e0295109. doi: 10.1371/journal.pone.0295109 (PMC11090294; doi:10.1371/journal.pone.0295109)
Supplement: S3 Table — A and D represent additive and dominance effects. (PDF) [file pone.0295109.s003.pdf]

S3 Table. Significant effects of the NOIA model. *A and D represent additive and dominance effects.*

gga1_168m	gga1_171m	gga1_171v	gga1_172v	gga1_174v	gga1_178v	Estimate	STD	p-value
A						-0.245	0.039	<0.001
					A	-0.093	0.034	0.006
D					A	0.218	0.082	0.008
D			A			0.313	0.132	0.018
D			D			-0.311	0.141	0.028
					D	0.099	0.045	0.028
	A			D		-0.275	0.132	0.037
A				D		-0.152	0.074	0.040
				D		0.119	0.059	0.046
				A	A	0.116	0.059	0.048
	D				D	-0.217	0.120	0.070
A			A			-0.145	0.080	0.071
		A			A	0.127	0.074	0.088
