# Supplementary material for: Complex genetic architecture of the chicken Growth1 QTL region
Source: PLoS One. 2024 May 13;19(5):e0295109. doi: 10.1371/journal.pone.0295109 (PMC11090294; doi:10.1371/journal.pone.0295109)
Supplement: S4 Table — Top 30 markers out of 1029 that passed the threshold. These markers were sorted based on their GWAS P-value and had a minimum MAF of 0.1 and a PhyloP score in the top 5% across the genome. (PDF) [file pone.0295109.s004.pdf]

S4 Table. Candidate markers. *Top 30 markers out of 1029 that passed the threshold. These markers were sorted based on their GWAS P-value and had a minimum MAF of 0.1 and a PhyloP score in the top 5% across the genome.*

POS	REF	ALT	GWAS	PhyloP	AAF	Effect	GeneName
168203470	A	T	16.61	2.84	0.23	intron_variant	ENOX1
168203263	A	C	16.61	1.72	0.23	intron_variant	ENOX1
170835310	A	G	16.22	1.32	0.48	upstream_gene_variant	ENSGALG00000050514
170835310	A	G	16.22	1.32	0.48	intergenic_region	ENSGALG00000050514-ENSGALG00000053256
168216902	G	T	16.21	1.22	0.22	intron_variant	ENOX1
170812758	T	C	16.17	1.24	0.5	intron_variant	ENSGALG00000050514
170808018	T	C	16.06	2.58	0.49	intron_variant	ENSGALG00000050514
168211327	A	G	16.01	1.37	0.22	intron_variant	ENOX1
168228062	A	G	15.98	1.41	0.23	intron_variant	ENOX1
168223991	T	C	15.89	2.12	0.23	intron_variant	ENOX1
168145231	G	A	15.78	1.21	0.22	intron_variant	ENOX1
170807834	T	G	15.68	1.51	0.48	intron_variant	ENSGALG00000050514
168185632	G	A	15.56	1.95	0.22	intron_variant	ENOX1
168178667	A	G	15.51	1.33	0.23	intron_variant	ENOX1
170755989	G	A	15.47	1.47	0.47	downstream_gene_variant	ENSGALG00000052226
170755989	G	A	15.47	1.47	0.47	downstream_gene_variant	ENSGALG00000052226
170755989	G	A	15.47	1.47	0.47	intergenic_region	ENSGALG00000052226-ENSGALG00000050514
170758053	C	A	15.45	1.31	0.47	downstream_gene_variant	ENSGALG00000052226
170758053	C	A	15.45	1.31	0.47	intergenic_region	ENSGALG00000052226-ENSGALG00000050514
170758053	C	A	15.45	1.31	0.47	downstream_gene_variant	ENSGALG00000052226
168138378	C	A	15.44	1.68	0.22	intron_variant	ENOX1
168402921	C	T	15.42	1.38	0.22	intron_variant	ENOX1

170756013	A	G	15.39	1.27	0.47	intergenic_region	ENSGALG00000052226-ENSGALG00000050514
170756013	A	G	15.39	1.27	0.47	downstream_gene_variant	ENSGALG00000052226
170756013	A	G	15.39	1.27	0.47	downstream_gene_variant	ENSGALG00000052226
168256753	G	A	15.33	1.46	0.23	intron_variant	ENOX1
168250829	C	A	15.31	1.88	0.23	intron_variant	ENOX1
167884153	T	C	15.3	1.45	0.23	intron_variant	ENSGALG00000052911
168282764	G	A	15.18	2.83	0.22	intron_variant	ENOX1
168342667	A	G	15.17	2.52	0.22	intron_variant	ENOX1
