# Supplementary material for: Complex genetic architecture of the chicken Growth1 QTL region
Source: PLoS One. 2024 May 13;19(5):e0295109. doi: 10.1371/journal.pone.0295109 (PMC11090294; doi:10.1371/journal.pone.0295109)
Supplement: S5 Table — Missense variants with the top 5% PhyloP score were sorted by GWAS significance (PDF) [file pone.0295109.s005.pdf]

S5 Table. Missense variants. *Missense variants with the top 5% PhyloP score were sorted by GWAS significance.*

POS	REF	ALT	GWAS	PhyloP	AAF	GeneName	HGVSc	HGVSp	SIFT
170685654	G	T	14.93	1.3	0.38	SPRYD7	c.47C>A	p.Ala16Asp	tolerated_low_confidence(0.07)
169127401	C	G	10.32	3.74	0.21	COG3	c.1489C>G	p.Gln497Glu	tolerated(0.54)
167844491	T	C	9.44	2.14	0.19	TNFSF11	c.281T>C	p.Ile94Thr	deleterious_low_confidence(0)
168961702	C	T	8.3	1.99	0.28	GPALPP1	c.440C>T	p.Thr147Ile	deleterious(0.04)
168962167	A	G	8.15	3.26	0.28	GPALPP1	c.677A>G	p.Lys226Arg	tolerated(0.08)
171246434	A	G	7.57	4.43	0.43	RNASEH2B	c.470A>G<br>c.467A>G	p.Tyr157Cys<br>p.Tyr156Cys	deleterious(0)
171405714	G	A	7.29	3.25	0.43	SERPINE3	c.530G>A	p.Arg177Gln	tolerated(1)
171411240	C	T	7.22	2.6	0.43	SERPINE3	c.920C>T	p.Ala307Val	deleterious(0.02)
167751114	G	A	6.23	3.84	0.19	AKAP11	c.5336G>A	p.Ser1779Asn	tolerated(0.57)
175921491	A	G	5.06	1.3	0.13	BRCA2	c.4879T>C	p.Tyr1627His	tolerated(0.07)
167181239	G	T	4.9	4.32	0.46	WBP4	c.535G>T	p.Ala179Ser	tolerated(0.22)
171648346	G	C	4.68	1.2	0.06	ATP7B	c.3354C>G<br>c.3564C>G	p.Ser1118Arg<br>p.Ser1188Arg	tolerated_low_confidence(0.09)
174699122	T	A	3.51	1.83	0.44	NBEA	c.3389A>T	p.Glu1130Val	deleterious_low_confidence(0)
171730823	G	A	3.35	1.31	0.41	CKAP2	c.1013C>T	p.Pro338Leu	tolerated(0.25)
167275900	C	T	3.09	1.53	0.28	ENSGALG00000032808	c.1550C>T	p.Ala517Val	tolerated_low_confidence(0.16)
172703819	C	T	3.08	4.72	0.18	FREM2	c.5734G>A	p.Val1912Ile	deleterious(0.01)
172692708	C	T	3.07	6.35	0.18	FREM2	c.6058G>A	p.Gly2020Ser	deleterious(0.03)
176297310	A	G	2.93	3.82	0.34	B3GLCT	c.467T>C	p.Met156Thr	tolerated_low_confidence(1)
172628436	T	A	2.64	1.89	0.18	ENSGALG00000017040	c.1469A>T	p.Tyr490Phe	tolerated(0.18)
176360253	G	C	2.03	2.48	0.81	HSPH1	c.1721G>C<br>c.1667G>C	p.Ser574Thr<br>p.Ser556Thr	tolerated_low_confidence(0.19)
177132509	G	C	1.72	2.4	0.24	POMP	c.25C>G	p.Leu9Val	deleterious(0.03)
176431951	C	G	1.67	3.32	0.24	USPL1	c.2635G>C<br>c.1570G>C	p.Glu879Gln<br>p.Glu524Gln	deleterious(0.01)
