# Supplementary material for: Complex genetic architecture of the chicken Growth1 QTL region
Source: PLoS One. 2024 May 13;19(5):e0295109. doi: 10.1371/journal.pone.0295109 (PMC11090294; doi:10.1371/journal.pone.0295109)
Supplement: S1 Fig — The y-axis shows the significance of the association study by negative log P-value. The strongest significance signals lay in the Growth1 region. (PDF) [file pone.0295109.s006.pdf]

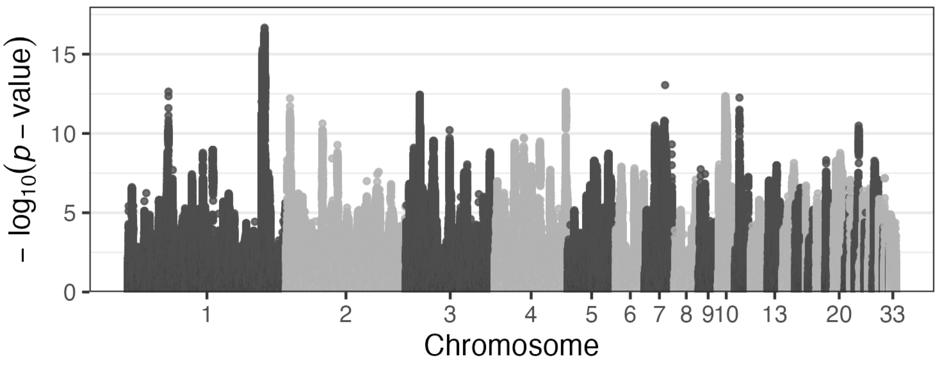

S1 Fig. Genome-wide association study. *The y-axis shows the significance of the association study by negative log p-value. The strongest significance signals lay in the Growth1 region.*
