# Supplementary material for: Complex genetic architecture of the chicken Growth1 QTL region
Source: PLoS One. 2024 May 13;19(5):e0295109. doi: 10.1371/journal.pone.0295109 (PMC11090294; doi:10.1371/journal.pone.0295109)
Supplement: S2 Fig — The result shows a 97% average agreement of heterozygous and 94% agreement of homozygous between GoldenGate assay and imputed genotype of F15 samples. (PDF) [file pone.0295109.s007.pdf]

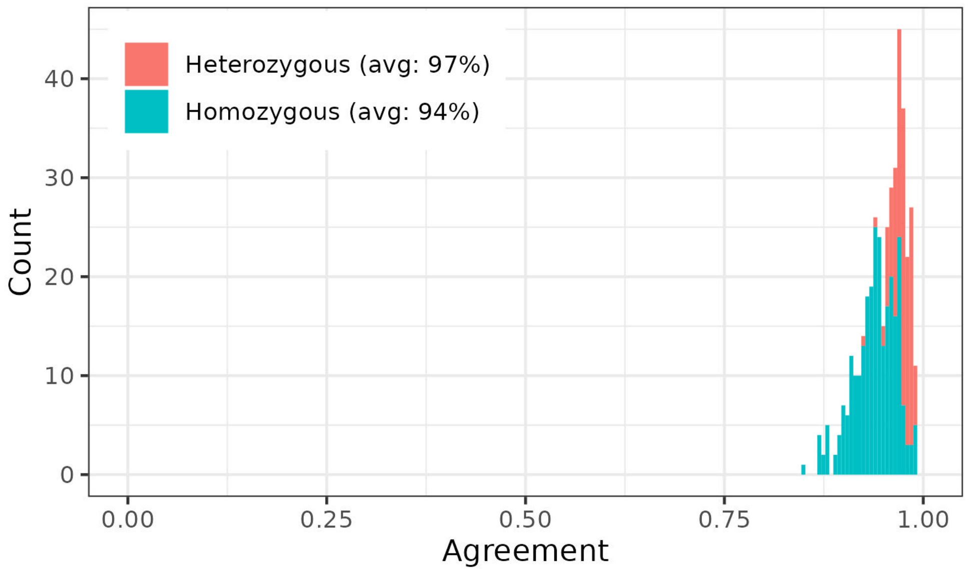

S2 Fig. Agreement between GoldenGate assay and imputed genotype. The result shows a 97% average agreement of heterozygous and 94% agreement of homozygous between GoldenGate assay and imputed genotype of F15 samples.
