# Supplementary material for: Complex genetic architecture of the chicken Growth1 QTL region
Source: PLoS One. 2024 May 13;19(5):e0295109. doi: 10.1371/journal.pone.0295109 (PMC11090294; doi:10.1371/journal.pone.0295109)
Supplement: S4 Fig — Figure (a), samples were grouped by the genotype of gga1_171m (red vertical line), in which 0, 1, and 2 represent the number of alternative alleles. Figure (b) shows the normalized average and standard deviation body weight in different conditions. The top SNP marker is annotated by the blue vertical line in Figure (a). (PDF) [file pone.0295109.s009.pdf]

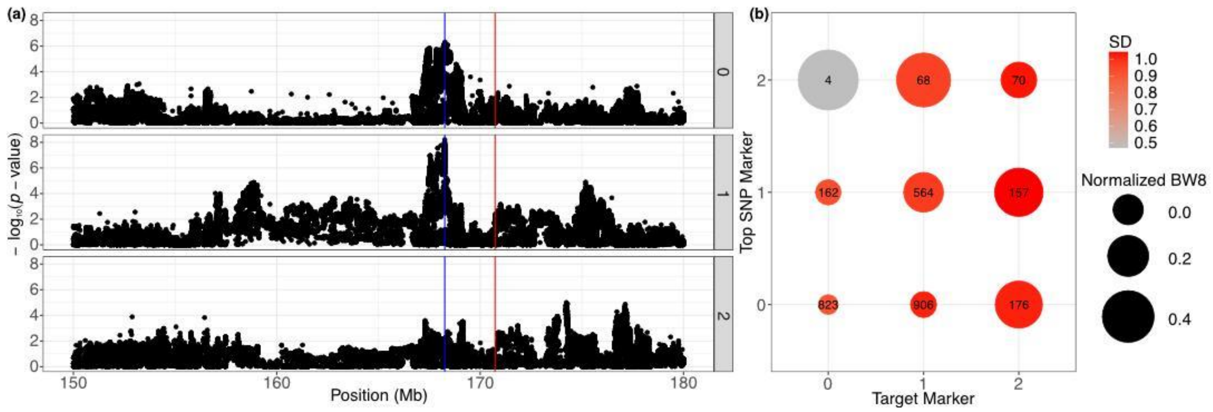

S4 Fig. Epistasis effects conditioned on gga1_171m. *Figure (a), samples were grouped by the genotype of gga1_171m (red vertical line), in which 0, 1, and 2 represent the number of alternative alleles. Figure (b) shows the normalized average and standard deviation body weight in different conditions. The top SNP marker is annotated by the blue vertical line in Figure (a).*
