# Supplementary material for: Complex genetic architecture of the chicken Growth1 QTL region
Source: PLoS One. 2024 May 13;19(5):e0295109. doi: 10.1371/journal.pone.0295109 (PMC11090294; doi:10.1371/journal.pone.0295109)
Supplement: S8 Fig — The y-axis shows the PhyloP score, and the x-axis shows the significance of the GWAS result. The dot size indicates the minor allele frequency. (PDF) [file pone.0295109.s013.pdf]

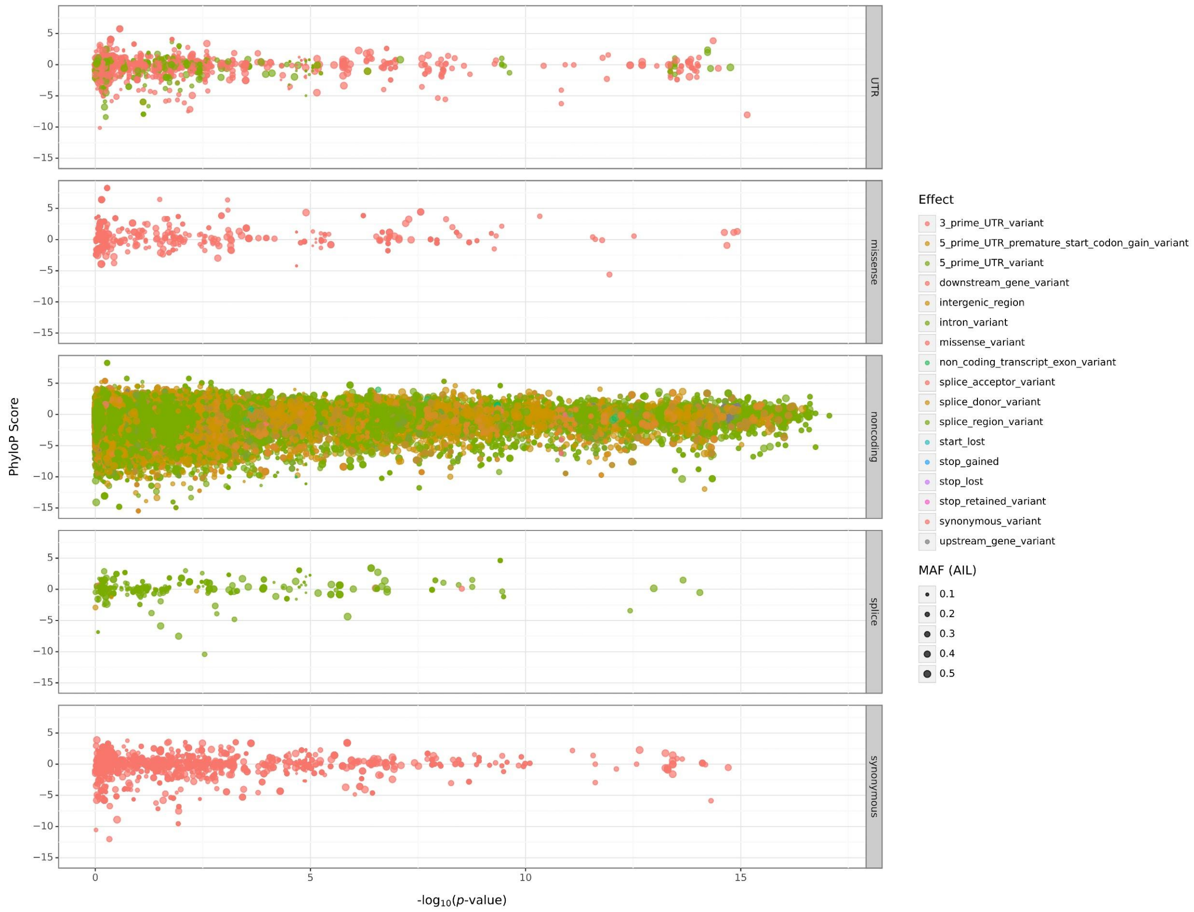

S8 Fig. SNPs on chromosome 1 150-180Mb region colored by sequence ontology terms. *The y-axis shows the PhyloP score, and the x-axis shows the significance of the GWAS result. The dot size indicates the minor allele frequency.*
