# Supplementary material for: Complex genetic architecture of the chicken Growth1 QTL region
Source: PLoS One. 2024 May 13;19(5):e0295109. doi: 10.1371/journal.pone.0295109 (PMC11090294; doi:10.1371/journal.pone.0295109)
Supplement: S9 Fig — Markers simultaneously satisfy the top 5% GWAS and PhyloP score threshold near the ribonuclease H2 subunit B (RNASEH2B) gene (annotated by orange background). (PDF) [file pone.0295109.s014.pdf]

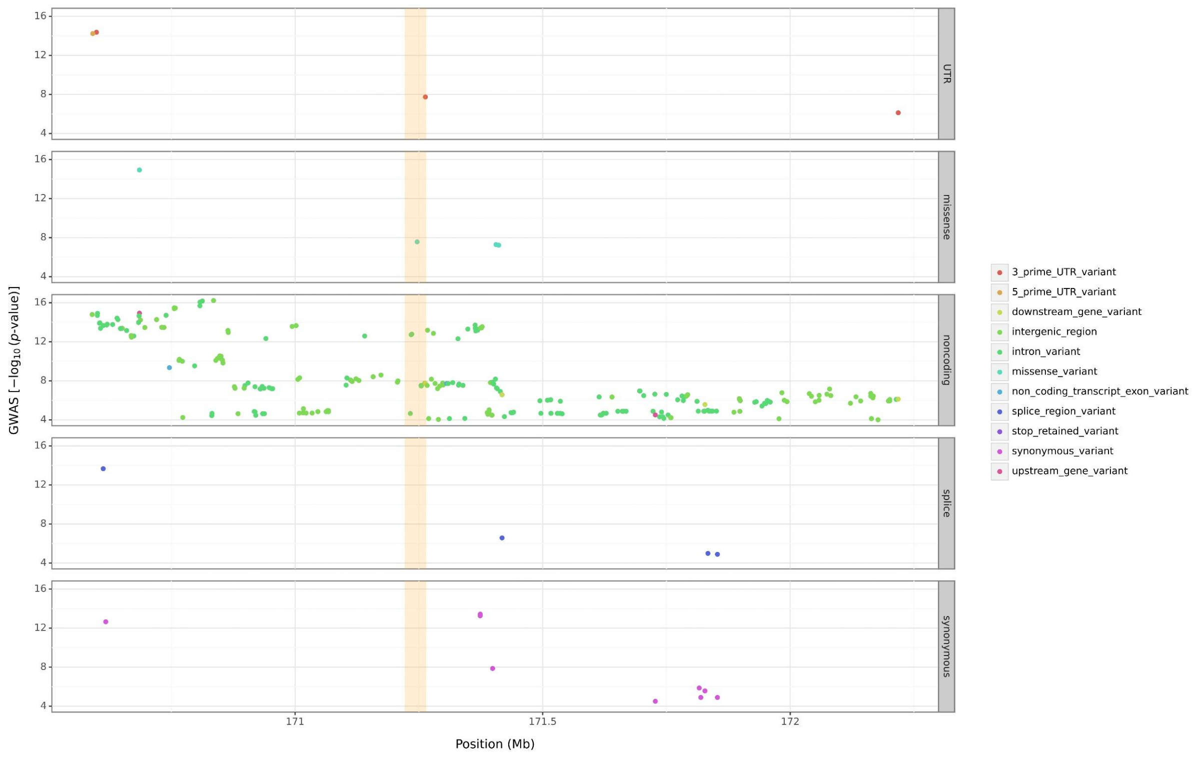

S9 Fig. Markers nearby RNASEH2B gene. *Markers simultaneously satisfy the top 5% GWAS and PhyloP score threshold near the ribonuclease H2 subunit B (RNASEH2B) gene (annotated by orange background).*
